# Draft genome sequence of *Streptomyces* sp. OS603R isolated from holy basil roots with promising antimicrobial and antitumor potential

**DOI:** 10.1128/MRA.00332-23

**Published:** 2023-09-11

**Authors:** Wuttichai Mhuantong, Wongsakorn Kwandee, Sirirat Boondireke, Darika Kongrit

**Affiliations:** 1 Enzyme Technology Research Team, National Center for Genetic Engineering and Biotechnology, Pathum Thani, Thailand; 2 Faculty of Innovative Agriculture and Fishery Establishment Project, Prince of Songkla University (Surat Thani campus), Surat Thani, Thailand; 3 Department of Stomatology, Faculty of Dentistry, Srinakharinwirot University, Bangkok, Thailand; 4 Faculty of Innovative Agriculture and Fishery Establishment Project, Prince of Songkla University (Surat Thani campus), Surat Thani, Thailand; Montana State University, Bozeman, Montana, USA

**Keywords:** *Streptomyces*, microbial genome, antimicrobial activity, antitumor activity

## Abstract

We report the genome sequence of *Streptomyces* sp. OS603R, isolated from holy basil roots. The strain possesses genes potentially responsible for antimicrobial and antitumor agents. The genome assembly comprises 7,521,075 bps with 72.29% GC content. The genome provides the basis for studies involving genes related to relevant bioactive compounds.

## ANNOUNCEMENT

Over the past decade, streptomycetes from medicinal plants have emerged as promising sources of bioactive compounds, particularly antimicrobial and antitumor agents (1–8). This is partly because they are relatively unexploited sources (1). Another reason is the possible genetic exchange between medicinal plants and endophytic streptomycetes, yielding similar bioactive substances to those of their hosts, as medicinal plants are abundant sources (2, 3). The genomic sequence of *Streptomyces* sp. OS603R was investigated to understand the biosynthetic gene clusters (BGCs) encoding these bioactive compounds.

OS603R was isolated from the roots of the holy basil (*Ocimum sanctum*) growing in Surat Thani Province, Southern Thailand (8°44'29.8"N 99°00'07.0"E). The strain was isolated using rifampin-resistant screening as follows: the root samples were collected from an agricultural farm. They were washed using running tap water, 2% sodium hypochlorite, rinsed with sterile water supplemented with fluconazole (32 µg/mL) and nystatin (100 µg/mL), and placed on *Streptomyces* Isolation Medium (SIM) supplemented with fluconazole (16 µg/mL), nystatin (50 µg/mL), nalidixic acid (20 µg/mL), and trimethoprim (20 µg/mL). The plate was kept at 30°C for 2 weeks. Each root was removed from the plate, mixed with sterile soil particles, and kept in a glass desiccator for 24 hours at 30°C. They were mixed and diluted with sterile water (1:1,000). Aliquots (0.2 mL) were placed on SIM supplemented with rifampin (20 µg/mL) in addition to the same antibiotics previously described and incubated at 30°C for 2 weeks. Control samples were prepared in the similar fashion as the samples but without rifampin supplementation (9, 10). The acquired rifampin-resistant isolates were re-purified on Bennett’s agar (11), preserved in 25% glycerol and stored at −80°C. Preliminary screening using agar plug and cytotoxicity assays was employed to identify potential antimicrobial and antitumor producers among the acquired isolates. Among them, OS603R exhibited the highest biological activities (data not shown).

The strain was cultured in Bennett’s broth (11) for 2 days at 30°C with shaking at 200 rpm. The genomic DNA was prepared using the GF-1 bacterial DNA extraction kit (Vivantis, Malaysia). All procedures were performed according to the manufacturer’s instructions. The NEBNext Ultra II DNA library Prep Kit (NEB, USA) was employed to prepare the genomic DNA libraries.

The genome sequencing was performed using the Illumina HiSeq platform [HE150 base pairs (bps)]. A total of 8,680,042 pairs of raw sequences were cleaned by removing low-quality sequences and trimming the adapter sequences with FASTP version number 0.20.0 (12). The cleaned sequences were assembled using SPAdes version 3.10.1 (13) with a minimum contig size of 500 bps. The contiguity of the assembled sequences was assessed using QUAST version number 5.0.1 (14). Genome completeness and contamination were evaluated using CheckM (15). Gene completeness of the genome assembly was determined using BUSCO version 5.4.2 (16) against “streptomycetales_odb10” in the OrthoDB database (17). The genome annotation was performed with the NCBI Prokaryotic Genome Annotation Pipeline (PGAP) version 6.5 (18). The OS603R BGCs were predicted using antiSMASH version 6.0 (19), which was implemented using the default options, “loose” detection strictness and “All on” extra features. A comprehensive overview of the genome assembly and annotation statistics is shown in Table 1.

**TABLE 1:** Genome sequencing statistics

Genome assembly		
Genome size	7.5 Mb	
Total ungapped length	7.5 Mb	
Number of scaffolds	82	
Scaffold N50	208.8 kb	
Scaffold L50	13	
Number of contigs	89	
Contig N50	179.8 kb	
Contig L50	15	
GC percent	72	
Genome coverage	341.3 x	
**Assessment of genome completeness and contamination by CheckM**		
Completeness	100%	
Contamination	0.22%	
**Assessment of gene completeness by BUSCO (streptomycetales_odb10)**		
Complete and single-copy BUSCOs (S)	1,569	
Complete and duplicated BUSCOs (D)	5	
Fragmented BUSCOs (F)	4	
Missing BUSCOs (M)	1	
**Genome annotation by PGAP version 6.5**		
# Genes	6,883	
# Protein-coding	6,578	
#Non-coding	79	

Further analysis of OS603R BGCs using antiSMASH revealed 20 regions of secondary metabolite BGCs, including two non-ribosomal peptide synthetases, three polyketide synthases, four ribosomally synthesized and post-translationally modified peptides, four terpenes, and two hybrid BGCs.

## Data Availability

This Whole Genome Shotgun project has been deposited in GenBank under the accession number JARKNF000000000. The version described in this paper is the first version, JARKNF010000000. The data are available under the NBCI BioProject accession number PRJNA945198. Raw reads have been deposited in NCBI’s SRA database with accession number SRR23934855.

## References

[1] Ma A, Jiang K, Chen B, Chen S, Qi X, Lu H, Liu J, Zhou X, Gao T, Li J, Zhao C. 2021. Evaluation of the anticarcinogenic potential of the endophyte, Streptomyces sp. LRE541 isolated from Lilium davidii var. Unicolor (hoog) cotton. Microb Cell Fact 20:217. doi:10.1186/s12934-021-01706-z 34863154 PMC8643024

[2] Golinska P, Wypij M, Agarkar G, Rathod D, Dahm H, Rai M. 2015. Endophytic actinobacteria of medicinal plants: diversity and bioactivity. Antonie Van Leeuwenhoek 108:267–289. doi:10.1007/s10482-015-0502-7 26093915 PMC4491368

[3] Khieu TN, Liu MJ, Nimaichand S, Quach NT, Chu-Ky S, Phi QT, Vu TT, Nguyen TD, Xiong Z, Prabhu DM, Li WJ. 2015. Characterization and evaluation of antimicrobial and cytotoxic effects of Streptomyces sp. HUST012 isolated from medicinal plant Dracaena cochinchinensis lour. Front Microbiol 6:574. doi:10.3389/fmicb.2015.00574 26106377 PMC4458686

[4] Remsing LL, González AM, Nur-e-Alam M, Fernández-Lozano MJ, Braña AF, Rix U, Oliveira MA, Méndez C, Salas JA, Rohr J. 2003. Mithramycin SK, a novel antitumor drug with improved therapeutic index, mithramycin SA, and demycarosyl-mithramycin SK three new products generated in the mithramycin producer Streptomyces argillaceus through combinatorial biosynthesis’’. J Am Chem Soc 125:5745–5753. doi:10.1021/ja034162h 12733914 PMC4480629

[5] Taechowisan T, Lu C, Shen Y, Lumyong S. 2007. Antitumor activity of 4-arylcoumarins from endophytic Streptomyces aureofaciens CMUAc130. J Cancer Res Ther 3:86–91. doi:10.4103/0973-1482.34685 17998729

[6] Wang L, Qiu P, Long X-F, Zhang S, Zeng Z-G, Tian Y-Q. 2016. Comparative analysis of chemical constituents, antimicrobial and antioxidant activities of ethylacetate extracts of Polygonum cuspidatum and its endophytic actinomycete, Streptomyces sp. Chin J Nat Med 14:117–123. doi:10.1016/S1875-5364(16)60004-3 26968677

[7] Wu Y, Lu C, Qian X, Huang Y, Shen Y. 2009. Diversities within genotypes, bioactivity, and biosynthetic genes of endophytic actinomycetes isolated from three pharmaceutical plants. Curr Microbiol 59:475–482. doi:10.1007/s00284-009-9463-2 19657693

[8] Zhang R, Han X, Xia Z, Luo X, Wan C, Zhang L. 2017. Streptomyces uozhongensis sp nov., a novel actinomycete with antifungal activity and antibacterial activity. Antonie Van Leeuwenhoek 110:195–203. doi:10.1007/s10482-016-0790-6 27752797

[9] Matsukawa E, Nakagawa Y, Iimura Y, Hayakawa M. 2007. A new enrichment method for the selective isolation of streptomycetes from the root surfaces of herbaceous plants. Actinomycetologica 21:66–69. doi:10.3209/saj.SAJ210202

[10] Thaker MN, Wang W, Spanogiannopoulos P, Waglechner N, King AM, Medina R, Wright GD. 2013. Identifying producers of antibacterial compounds by screening for antibiotic resistance. Nat Biotechnol 31:922–927. doi:10.1038/nbt.2685 24056948

[11] Thaker MN, Waglechner N, Wright GD. 2014. Antibiotic resistance-mediated isolation of scaffold-specific natural product producers. Nat Protoc 9:1469–1479. doi:10.1038/nprot.2014.093 24874813

[12] Chen S, Zhou Y, Chen Y, Gu J. 2018. Fastp: an ultra-fast all-in-one FASTQ preprocessor. Bioinformatics 34:i884–i890. doi:10.1093/bioinformatics/bty560 30423086 PMC6129281

[13] Prjibelski A, Antipov D, Meleshko D, Lapidus A, Korobeynikov A. 2020. Using the SPAdes de novo assembler. Curr Protoc Bioinformatics 70:e102. doi:10.1002/cpbi.102 32559359

[14] Gurevich A, Saveliev V, Vyahhi N, Tesler G. 2013. QUAST: quality assessment tool for genome assemblies. Bioinformatics 29:1072–1075. doi:10.1093/bioinformatics/btt086 23422339 PMC3624806

[15] Parks DH, Imelfort M, Skennerton CT, Hugenholtz P, Tyson GW. 2015. Checkm: assessing the quality of microbial genomes recovered from isolates, single cells, and metagenomes. Genome Res. 25:1043–1055. doi:10.1101/gr.186072.114 25977477 PMC4484387

[16] Manni M, Berkeley MR, Seppey M, Zdobnov EM. 2021. BUSCO: assessing genomic data quality and beyond. Curr Protoc 1:e323. doi:10.1002/cpz1.323 34936221

[17] Kriventseva EV, Kuznetsov D, Tegenfeldt F, Manni M, Dias R, Simão FA, Zdobnov EM. 2019. OrthoDB v10: sampling the diversity of animal, plant, fungal, protist, bacterial and viral genomes for evolutionary and functional annotations of orthologs. Nucleic Acids Res 47:D807–D811. doi:10.1093/nar/gky1053 30395283 PMC6323947

[18] Tatusova T, DiCuccio M, Badretdin A, Chetvernin V, Nawrocki EP, Zaslavsky L, Lomsadze A, Pruitt KD, Borodovsky M, Ostell J. 2016. NCBI prokaryotic genome annotation pipeline. Nucleic Acids Res 44:6614–6624. doi:10.1093/nar/gkw569 27342282 PMC5001611

[19] Blin K, Shaw S, Kloosterman AM, Charlop-Powers Z, van Wezel GP, Medema MH, Weber T. 2021. AntiSMASH 6.0: improving cluster detection and comparison capabilities. Nucleic Acids Res 49:W29–W35. doi:10.1093/nar/gkab335 33978755 PMC8262755

